# Effect of Mixing Technology on Homogeneity and Quality of Sodium Naproxen Tablets: Technological and Analytical Evaluation Using HPLC Method

**DOI:** 10.3390/molecules30153119

**Published:** 2025-07-25

**Authors:** Mateusz Przywara, Regina Lech-Przywara, Patrycja Rupar, Wojciech Zapała

**Affiliations:** 1 Department of Chemical and Process Engineering, Rzeszow University of Technology, al. Powstańców Warszawy 6, 35-959 Rzeszów, Polandichwz@prz.edu.pl (W.Z.); 2 Doctoral School of the Rzeszow University of Technology, al. Powstańców Warszawy 12, 35-959 Rzeszów, Poland; d555@stud.prz.edu.pl

**Keywords:** naproxen sodium, content uniformity, powder mixing, direct compression, V-type mixer, ball mill, HPLC, tablet quality, analytical methods, pharmaceutical formulation

## Abstract

The uniform distribution of APIs is essential in tablet formulations, particularly in direct compression, where powder blending is the only means of ensuring dose homogeneity. This study evaluated the influence of three mixing techniques—V-type mixer, planetary ball mill, and vibratory ball mill—on the physical properties and content uniformity of naproxen sodium tablets. Blends consisting of naproxen sodium, cellulose, PVP, calcium carbonate, and magnesium stearate were prepared under varied mixing intensities and characterized in terms of flowability, compressibility, and particle size distribution. The resulting tablets were analyzed for weight, thickness, hardness, friability, and API content using a simplified bypass HPLC method. The V-type mixer yielded tablets with the most consistent weight and thickness, despite the poorest blend flow properties. Vibratory milling produced the hardest tablets and best API content uniformity, although high-energy processing introduced variability at longer mixing times. The analytical method proved fast and robust, allowing for reliable API quantification without full chromatographic separation. These findings underscore the need to balance mechanical blending energy with formulation properties and support the use of streamlined analytical strategies in pharmaceutical development.

## 1. Introduction

Achieving content uniformity in pharmaceutical tablet formulations remains one of the most fundamental requirements of product quality and regulatory compliance [1,2]. Especially in direct compression processes, where powders are compacted without a granulation step, ensuring homogeneity of the blend is critical for dose accuracy, therapeutic efficacy, and patient safety [3,4]. Direct compression is a cost-effective tableting method that requires less processing steps [5] and is suitable for drugs sensitive to temperature and moisture [6]. This technique involves producing tablets by compressing a powder mixture of the active ingredient and excipients directly, without prior granulation [5]. Tablets prepared by direct compression disintegrate into individual API particles rather than granules when exposed to dissolution fluids, resulting in faster dissolution [7]. The uniform distribution of the active pharmaceutical ingredient (API) within the powder mixture is influenced by several factors, including the physicochemical properties of the components and the blending technique employed. Inhomogeneity in powder mixtures can lead to drug content variability, particularly in low-dose formulations or those involving cohesive or poorly flowing APIs [8,9].

Powder blending remains a key unit operation in solid dosage manufacturing [10]. A wide range of mixing equipment is available, including tumbling mixers, planetary ball mills [11], and vibrating ball mills [12], each offering different mechanisms for dispersing ingredients. Previous studies have shown that mixer geometry, agitation speed, and blending time can substantially influence the degree of homogeneity achieved [8,13]. In this context, our study compares the performance of three commonly used mixing systems—V-type tumble mixer, planetary ball mill, and vibrating ball mill—on the uniformity and final quality of naproxen sodium tablets produced via direct compression. We evaluated both physical parameters (mass, thickness, friability, hardness) and chemical uniformity using a simplified quantitative method.

Naproxen sodium was selected as a model API due to its well-known analytical behavior and broad pharmaceutical application [14,15]. This API is characterized by well-documented poor flowability and low compressibility [2,14,16]. These properties pose significant challenges for formulation via direct compression. It is a nonsteroidal anti-inflammatory drug (NSAID) with favorable UV absorbance properties, which make it particularly suitable for spectrophotometric and chromatographic analyses. The model formulation also included excipients commonly used in tablet production: cellulose, polyvinylpyrrolidone (PVP), magnesium stearate, and calcium carbonate. These components were selected for their standard roles as filler, binder, lubricant, and diluent, respectively, representing a typical direct compression matrix.

The analysis of API content is an essential aspect of formulation development, quality assurance, and regulatory procedures. Although HPLC remains an important standard due to its high sensitivity, selectivity, and reproducibility, in simpler, impurity-free matrices, methods that bypass full chromatographic separation are increasingly being employed. For example, the Flow Injection–Chemiluminescence (FI-CL) technique has demonstrated the ability to detect naproxen at concentrations as low as 3.0 × 10^−8^ g/mL without using a column [17], while the Flow Injection Analysis with Ultraviolet detection FIA-UV approach has achieved high precision (RSD ≈ 2.2%) and a quantification limit of 5.8 × 10^−7^ M [15]. Amperometric determination using electrodes modified with carbon nanotubes enabled simultaneous analysis of naproxen and paracetamol, achieving throughputs of up to 90 analyses per hour [18], while employing a short C18 column (10 × 4 mm) allowed for complete separation of naproxen in under 5 min [19].

Advanced chemometric techniques, such as Classical Least Squares (CLS), Principal Component Regression (PCR), and Partial Least Squares (PLS), allow precise resolution of overlapping signals and simultaneous determination of components in complex matrices [20], while stability studies conducted according to International Council for Harmonisation (ICH) guidelines ensure reliable profiling of degradation products formed under acidic, basic, oxidative, or photolytic conditions [21]. Additional methods, such as differential thermal analysis using Thermogravimetric Analysis (TGA) and calculated Differential Thermal Analysis (c-DTA), are used for rapid differentiation between the acidic form and the sodium salt of naproxen in finished formulations [22], and High-Performance Liquid Chromatography (HPLC) solubility studies provide key data for formulation optimization [23].

From an environmental perspective, research into the electrochemical removal of naproxen from water has shown that modified anodes can achieve higher efficiency with lower energy consumption [24]. Simple quality control (QC) procedures based on RP-HPLC enable rapid batch release into production [25], while more advanced chromatographic strategies allow simultaneous profiling of potential impurities in combination drug products [26]. Optimization of the mixing process, together with meticulous selection of analytical tools—from rapid bypass HPLC techniques and chemometric algorithms to stability testing and thermal analysis—is crucial for ensuring high API uniformity and effective QC of pharmaceutical products.

In our research, we completely eliminated the use of a chromatographic column, employing only a bypass setup with UV detection. Thanks to the clear sample matrix and strong light absorption by naproxen, we achieved rapid (<2 min), precise, and cost-effective API determination, making this method particularly attractive for high-throughput laboratories and facilities with limited resources.

In summary, this study provides an integrated analytical and technological assessment of how different mixing techniques affect tablet quality and API uniformity. It also proposes a simplified but reliable method for sodium naproxen quantification, contributing to the ongoing development of more efficient and accessible pharmaceutical quality control strategies.

## 2. Results

As shown in Table 1, nine experimental batches were prepared using three mixing strategies. The first series (V1–V3) was mixed in a V-type blender at 10, 20, and 30 rpm for 20 min. The second series (PBM1–PBM3) was blended using a planetary ball mill operating at 200, 300, and 400 rpm for 5 min. The third series (VBM1–VBM3) was mixed using a vibratory ball mill for 2, 5, and 10 min, respectively. All batches used a consistent powder formulation containing sodium naproxen (API), PVP, magnesium stearate, cellulose, and calcium carbonate.

### 2.1. Raw Material Properties

**Figure 1:**
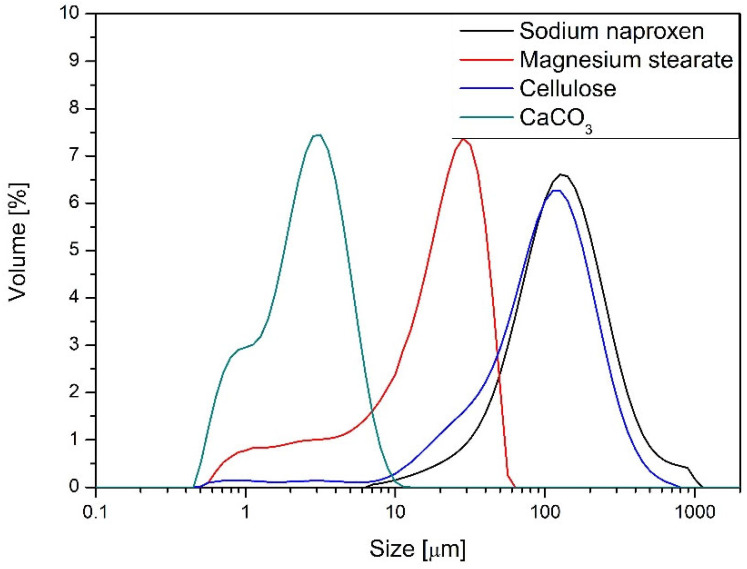
Particle size distribution of powder materials.

The physical characteristics of the raw materials used in this study—sodium naproxen, PVP, magnesium stearate, cellulose, and calcium carbonate—were assessed through standard powder flow and density tests. The data are summarized in Table 2 and Figure 1.

Sodium naproxen exhibited the poorest flow and packing behavior among all tested materials. Its angle of repose was 46.1°, indicating high cohesiveness and poor flowability. The compressibility index of 45.1% and Hausner ratio of 1.82 reflect significant densification upon tapping, suggesting poor bulk powder behavior during handling or blending. The difference between aerated and packed bulk density was substantial (0.380 vs. 0.692 g/cm^3^), further confirming poor packing efficiency. These properties classify sodium naproxen as a challenging API for direct compression. PVP showed the most favorable flow and compressibility profile. It had the lowest compressibility index (16.5%) and a Hausner ratio of 1.20, both indicative of excellent flow properties and minimal interparticle friction. Its angle of repose was 39.9°, which also falls within the acceptable range for freely flowing powders. These parameters justify the use of PVP as a suitable binder and carrier in powder blends aimed for direct compression. Magnesium stearate, a common lubricant, presented intermediate flow characteristics. It had a moderate angle of repose (39.7°) and compressibility index of 34.8%, resulting in a Hausner ratio of 1.53. These values suggest fair-to-poor flow and the potential to induce variability in blend homogeneity if not properly dispersed. Cellulose, widely used as a filler and disintegrant, demonstrated reasonable performance. With an angle of repose of 44.2°, compressibility of 18.4%, and a Hausner ratio of 1.23, it showed good bulk flow and compaction characteristics. Its behavior aligns well with its common use in direct compression formulations. Calcium carbonate had the highest bulk density among the excipients (packed: 0.864 g/cm^3^), but also the highest compressibility index (48.0%) and Hausner ratio (1.92), which indicate poor flow and pronounced packing inefficiency. Its angle of repose (43.1°) confirmed marginally cohesive behavior. The combination of high density and poor flow suggests calcium carbonate may segregate in blends or cause die-filling inconsistencies during compression. Among the analyzed substances, calcium carbonate exhibits the smallest particle size. The particles range from 0.5 μm to 11 μm, with those around 3 μm being the most prevalent. Cellulose shows a particle size distribution similar to that of sodium naproxen, a key component of the tablets. In both naproxen and cellulose, particles approximately 140 μm in size dominate. The particle size distribution (PSD) of magnesium stearate falls between that of calcium carbonate and the ground forms of sodium naproxen and cellulose. It has the widest range, extending from 0.5 μm to 79 μm, with particles around 25 μm making up the largest fraction. The PVP used in the formulation is reported by the manufacturer to have an average particle size close to 20 μm.

Overall, the powder characterization results underscore the importance of understanding the individual flow and packing behaviors of each component. The formulation includes both highly cohesive (naproxen, calcium carbonate) and free-flowing (PVP, cellulose) materials, which presents challenges for uniform blending. These disparities in flowability and compressibility are likely to affect the efficiency of mixing techniques, blend uniformity, and downstream tableting performance.

### 2.2. Blend Properties

The physical properties of powder blends used for tablet compression were analyzed to evaluate the impact of mixing technology on flow behavior and packing characteristics. The measured parameters include angle of repose, angle of fall, angle of spatula, bulk and tapped densities, compressibility index, and Hausner ratio. The results are summarized in Table 3.

**Figure 2:**
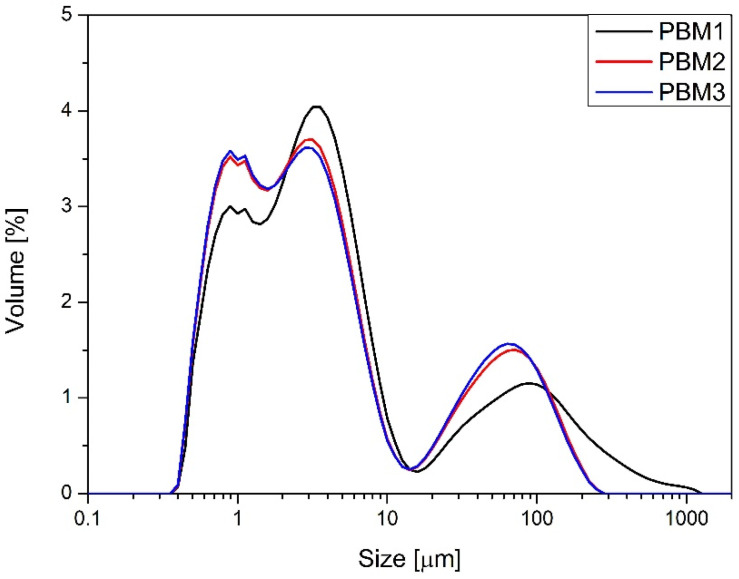
Particle size distribution of blends prepared in planetary ball mill.

**Figure 3:**
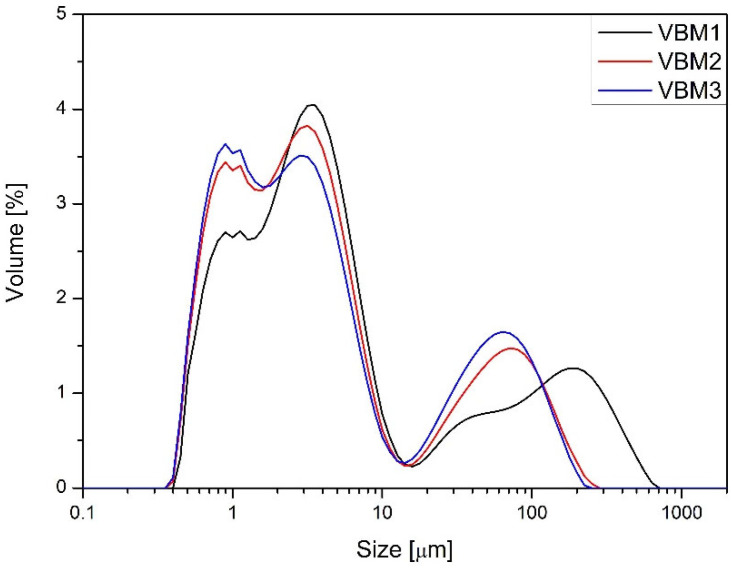
Particle size distribution of blends prepared in vibrating ball mill.

Blends prepared using the V-type mixer (V1–V3) demonstrated moderate-to-poor flowability. Their angles of repose ranged from 39.6° to 42.7°, and compressibility indices exceeded 43% in all cases, with Hausner ratios between 1.77 and 1.79. These values indicate highly compressible and relatively cohesive powders. Bulk densities ranged from 0.408 to 0.418 g/cm^3^, and packed densities approached 0.75 g/cm^3^, suggesting limited flow and a strong tendency for densification under tapping. The angle of spatula exceeded 55° in all V-type batches, further confirming their cohesive nature. Blends prepared using the planetary ball mill (PBM1–PBM3) exhibited slightly improved flow behavior and compressibility. Angles of repose ranged from 39.1° to 40.3°, with compressibility indices decreasing progressively from 41.7% (PBM1) to 38.7% (PBM3). The Hausner ratio decreased accordingly from 1.71 to 1.63. These improvements reflect the effect of mechanical energy input, which likely reduced particle size and improved powder packing. The bulk and tapped densities also increased slightly with mixing intensity, indicating better compaction potential. However, the angle of difference and angle of spatula remained comparable to V-type blends, suggesting only modest gains in flow. The vibratory ball mill blends (VBM1–VBM3) achieved the best flow and compressibility profiles. Angles of repose remained in the 39–41° range, but compressibility indices dropped significantly to 33.7–34.5%, with Hausner ratios as low as 1.51. These values indicate improved packing efficiency and reduced cohesiveness, likely due to particle size reduction and enhanced homogenization. The aerated and packed bulk densities for VBM3 reached 0.488 g/cm^3^ and 0.739 g/cm^3^, respectively—the highest among all tested blends. However, the increasing angle of difference in VBM2 and VBM3 (up to 25.6°) may point to blend instability or segregation tendencies under dynamic conditions. PSD was assessed for the PBM and VBM blends (Figure 2 and Figure 3). All milled samples showed relatively wide PSD profiles, with VBM2 and VBM3 in particular displaying signs of agglomeration and bimodal particle distributions or not homogeneous milling of the components of the mixture. These profiles indicate heterogeneous energy transfer during high-energy mixing, which may lead to local overprocessing or re-agglomeration. PSD was not evaluated for V-type mixer batches, as these underwent no milling or mechanical size reduction. In summary, flow and compressibility characteristics varied markedly depending on the mixing technology used. The V-type mixer produced blends with poor compressibility and flow, yet these were sufficient for tableting due to good component compatibility. Planetary and vibratory ball mills improved bulk behavior but introduced risks of segregation and blend instability, especially at higher energy levels. These findings align with observed differences in tablet mass, thickness, and API content uniformity, further emphasizing the influence of blend quality on downstream performance.

### 2.3. Tablet Properties

**Figure 4:**
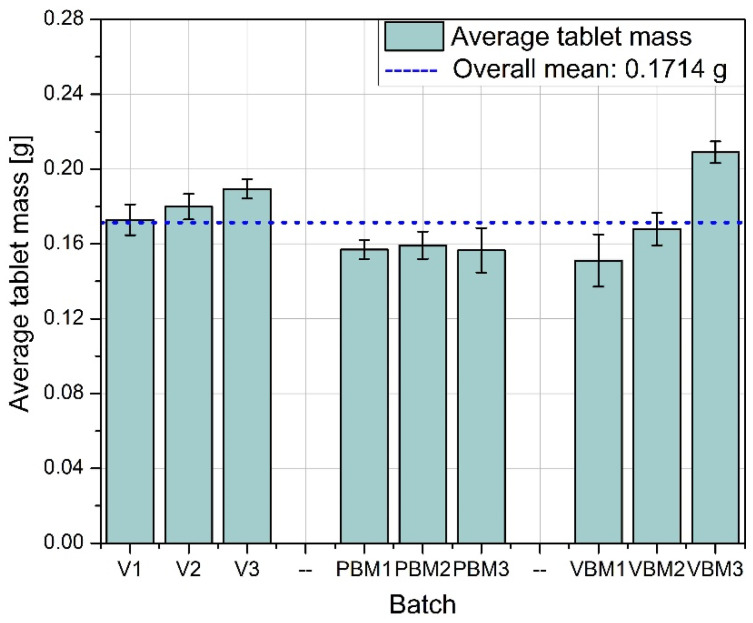
Average tablet mass.

Figure 4 shows the average tablet mass obtained for each of the mixing methods tested. Tablets produced from mixtures prepared in a V-type mixer (V1–V3) have the most stable and close to average weight values, especially in batches V2 and V3. This indicates good homogeneity of the mixture in terms of quantity, which translated into precise powder dosing during tableting. In the case of PBM (PBM1–PBM3), lower average tablet weights were observed in all batches compared to the V-mixer, which may indicate deteriorated mixture flowability or the effects of excessive powder grinding and segregation. The VBM (VBM1–VBM3) showed differential performance—while VBM1 and VBM2 achieved weights slightly above the global average, VBM3 significantly exceeded it, which may be due to local differences in density or instability in the dispensing process.

**Figure 5:**
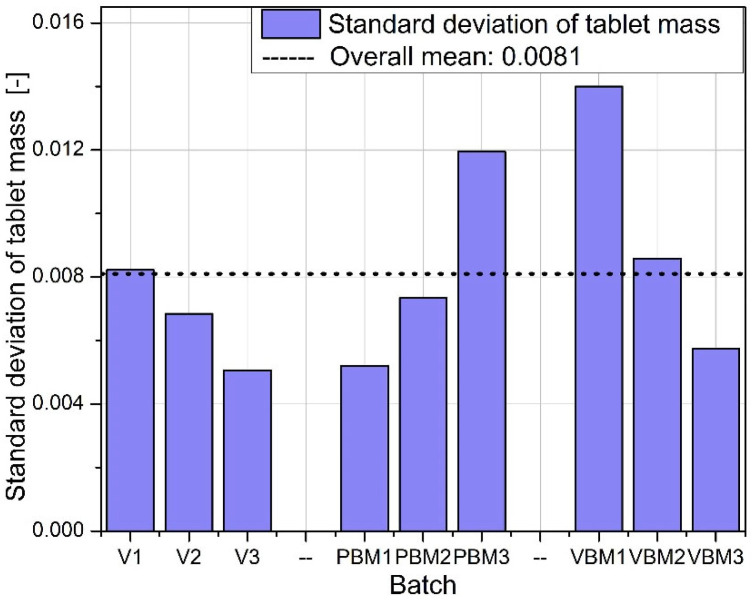
Standard deviation of tablet mass.

The standard deviations of tablet mass are shown in Figure 5. The lowest variability was achieved for tablets from batches V2 and V3, confirming the high repeatability of dosing for the V-type mixer. For PBMs, elevated variances are noticeable, especially in the PBM3 batch, indicating a problem with mixture homogeneity or flowability during die filling. VBM2 shows the highest weight variation of all batches tested, suggesting instability in the dosing process or uneven packing of the powder despite good mixing quality.

**Figure 6:**
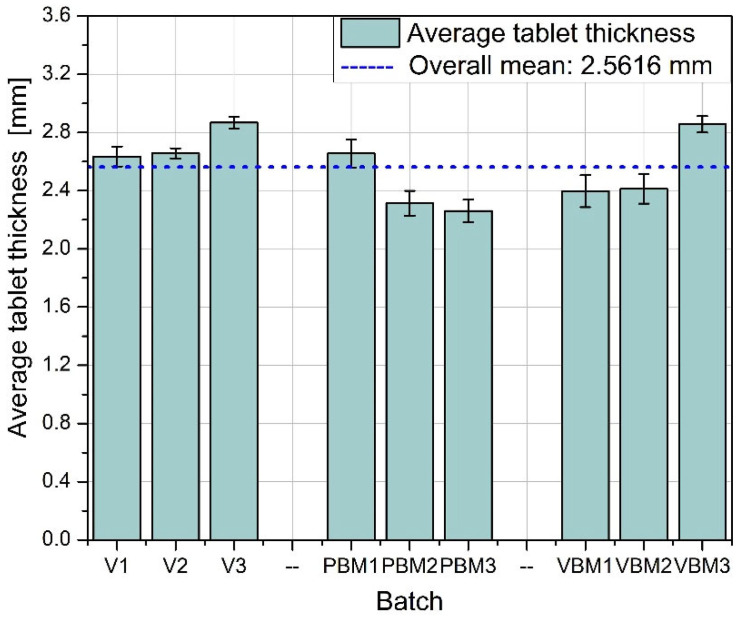
Average tablet thickness.

**Figure 7:**
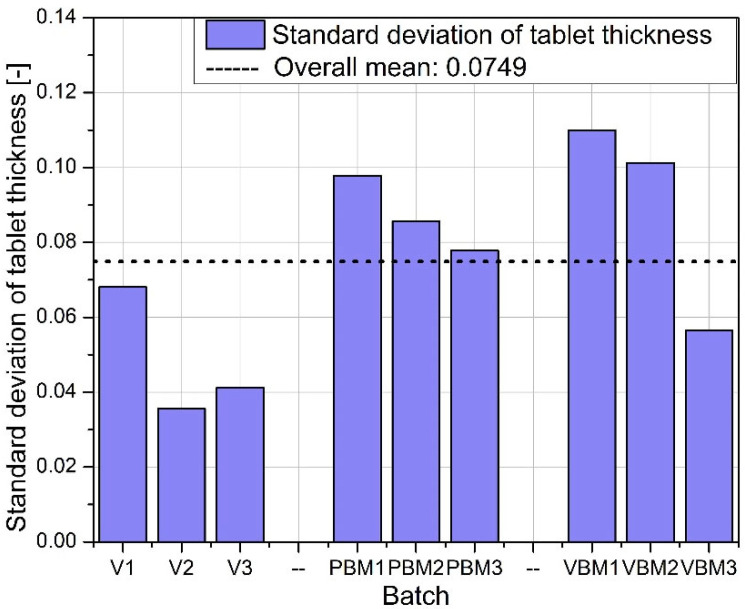
Standard deviation of tablet thickness.

Figure 6 shows the average thickness of tablets in the batches studied. The greatest thickness consistency is again observed in batches V1–V3, indicating good control of the pressing process and uniform compression of the mixture. PBM1 and PBM2 show a slightly lower average thickness, while PBM3 is close to the overall average. The variation in these values may indicate varying compactability of the powder or varying rheological properties of the mixtures depending on the grinding intensity. VBM3 reaches a thickness close to the average, while VBM1 and VBM2 are slightly below this level, suggesting possible differences in the degree of compression between batches. Figure 7 illustrates the variation in tablet thickness, reflecting the stability of the compression process. Batch V2 stands out for having the lowest standard deviation, confirming its exceptional repeatability in terms of thickness and overall tablet quality. PBM3 and VBM2, on the other hand, are characterized by the highest deviations, which may be the result of insufficient particle dispersion or instability in the bulk density of the mixtures during pressing.

**Figure 8:**
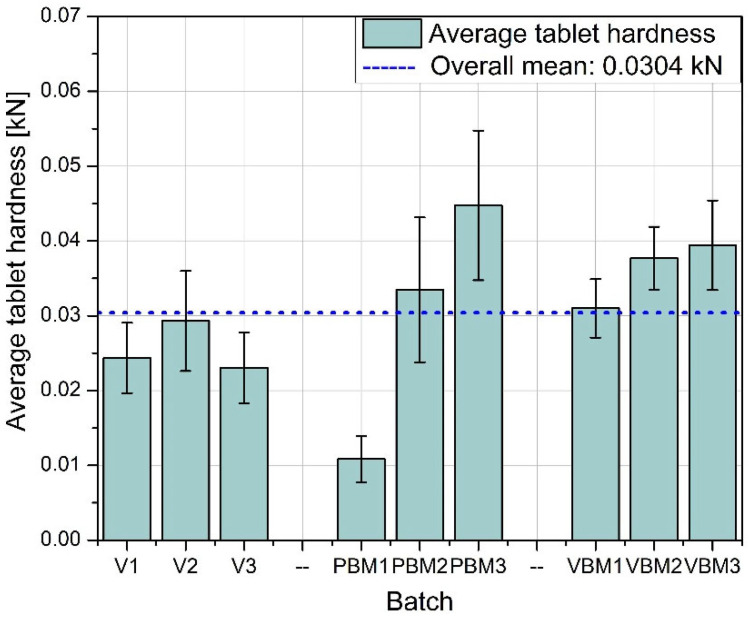
Average tablet hardness.

**Figure 9:**
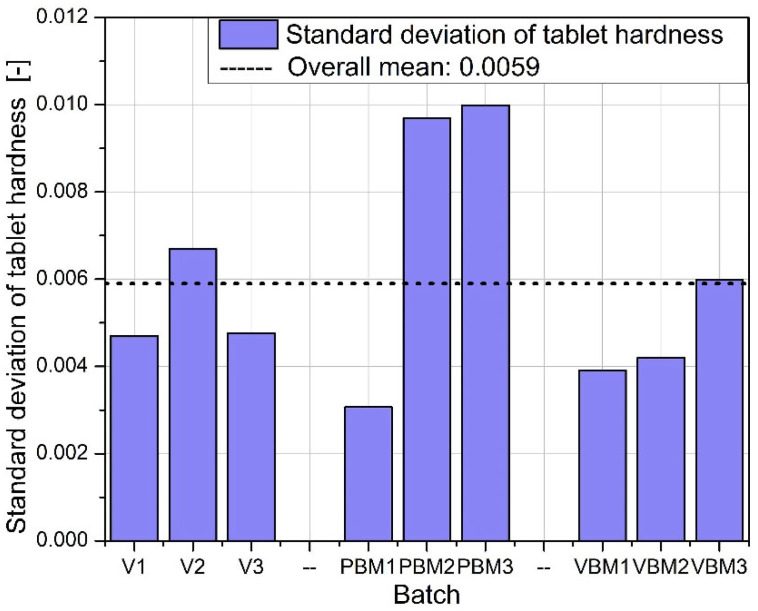
Standard deviation of tablet hardness.

**Figure 10:**
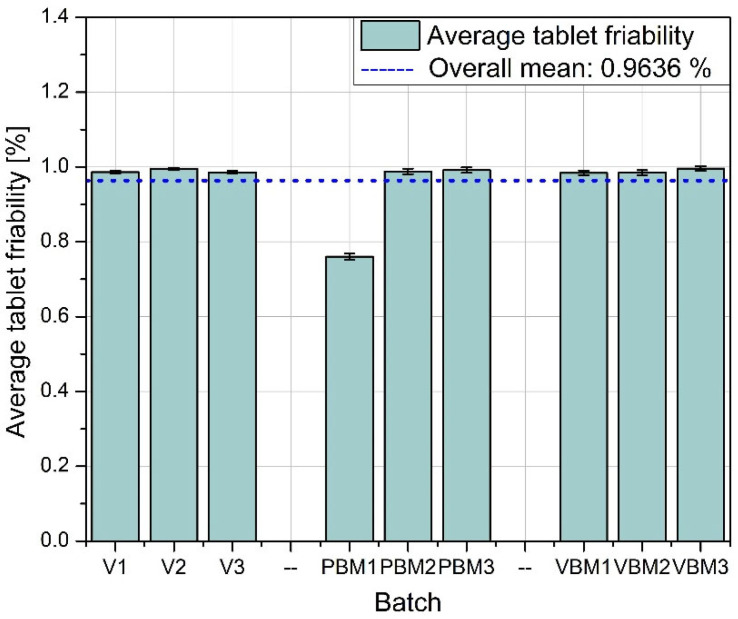
Average tablet friability.

**Figure 11:**
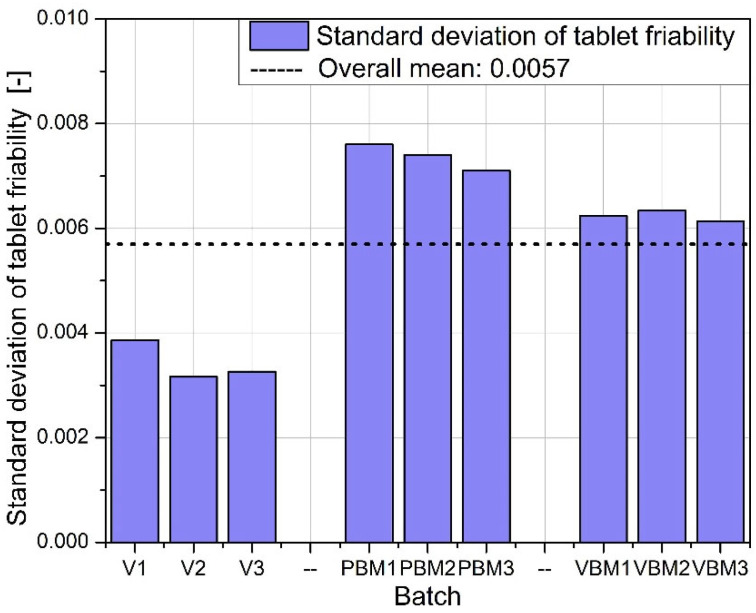
Standard deviation of tablet friability.

The physical uniformity of the tablets (mass, thickness) depends not only on the intensity of mixing but also on the control of the filling and pressing process, which is particularly important when using high-shear methods of compound preparation. The graphs in Figure 8, Figure 9, Figure 10 and Figure 11 show the results of hardness and friability measurements of tablets made from mixtures prepared by three different methods: a V-type mixer, a planetary ball mill (PBM), and a vibrating ball mill (VBM). These parameters illustrate the mechanical strength of the finished pharmaceutical forms and their resistance to physical damage.

Figure 8 shows the average hardness of the tablets obtained in each of the tested batches. The highest hardness values were achieved for tablets prepared using a vibrating ball mill (VBM1–VBM3), indicating good compactability and strong bonds between particles during pressing. PBM tablets show intermediate values, while the lowest hardness was obtained in tablets from the V-type mixer, suggesting weaker blend cohesion and lower packing density during compression. Figure 9 shows the standard deviation of tablet hardness, which provides a measure of the mechanical uniformity of tablets in each batch. The lowest variability is observed for the VBM batches, confirming their highest reproducibility and quality control. For PBM and V, the variability in hardness is moderate, which may be due to the heterogeneous distribution of active and auxiliary ingredients in the powder before tableting. Figure 10 shows the average friability of the tablets, i.e., their susceptibility to weight loss under mechanical loading conditions (e.g., during packaging or transportation). All three methods performed very similarly—the abrasion values remain at low, similar levels, with no clear differences between batches. These results indicate that regardless of the mixing technology used, the resulting tablets have acceptable abrasion resistance, meeting typical pharmacopeial requirements (<1%). Figure 11 shows the standard deviation of tablet abrasion. As with the mean values, the differences between batches are minimal, and the obtained deviations indicate good repeatability of the results. A slight qualitative advantage can be attributed to VBM, where the deviations are slightly lower, which may be due to the more homogeneous structure of the powder after mixing. A compilation of the results in Figure 8, Figure 9, Figure 10 and Figure 11 confirms that mixing by the vibratory ball mill (VBM) method leads to the most homogeneous and mechanically robust tablets. Tablets obtained by this method have both the highest hardness and the lowest variation in hardness and friability. The V-type mixer, on the other hand, despite its simplicity and popularity, generates the highest mechanical variability, which may be the result of limited mixing efficiency, especially for powders with poor flow and high cohesion. Average abrasion values remain low in all groups, suggesting that the tableting parameters used were appropriately chosen and produced forms with good resistance to physical damage, regardless of the mixture preparation method.

### 2.4. API Content

A simplified HPLC method, in which the chromatographic column was replaced by a bypass capillary junction, was used to rapidly assess the sodium naproxen content of tablet samples. This technique offers several advantages over conventional HPLC, including increased sensitivity, reduced solvent consumption, and higher peak concentrations at the detector. This approach was feasible due to the absence of interfering matrix components and the strong UV absorbance of sodium naproxen.

**Figure 12:**
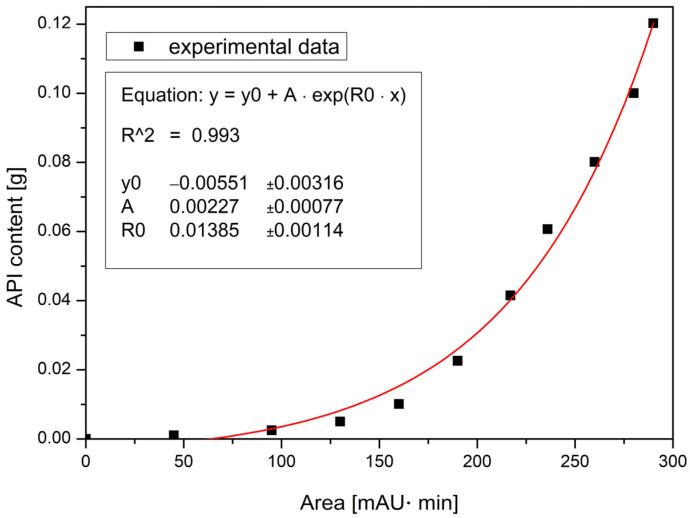
Calibration curve.

A calibration curve was constructed using ten standard solutions with known API contents of 0.001, 0.003, 0.005, 0.010, 0.023, 0.042, 0.061, 0.080, 0.100, and 0.120 g per sample. The resulting calibration curve was fitted using an exponential model, yielding a determination coefficient of R^2^ = 0.993 (Figure 12).

Given the development-oriented nature of this study and the need for efficient analysis of multiple batches, the bypass method provided time and resource savings. Although the method was not validated in full compliance with ICH Q2 guidelines, its reproducibility and speed made it suitable for comparative process evaluation. Therefore, the bypass method served as a rapid, resource-efficient alternative for assessing API uniformity across batches.

**Figure 13:**
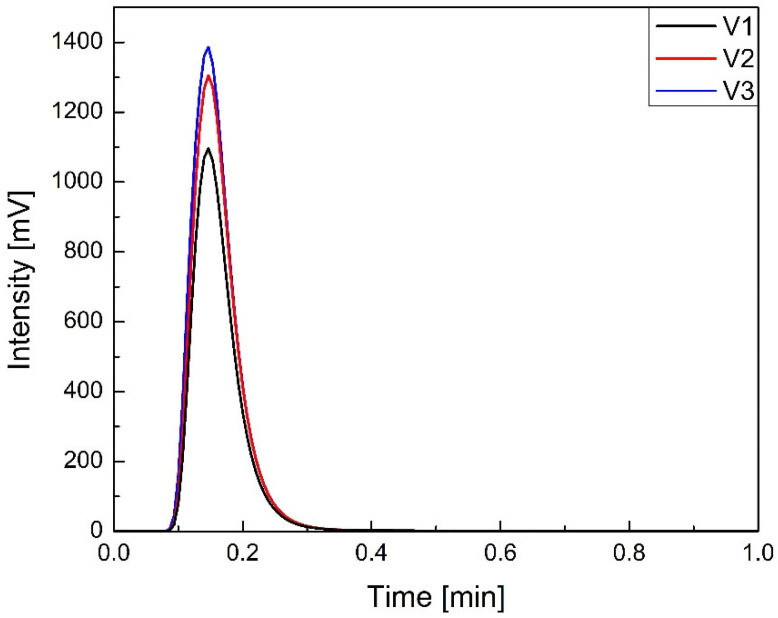
An example comparison of chromatograms obtained for mixtures V1–V3.

**Figure 14:**
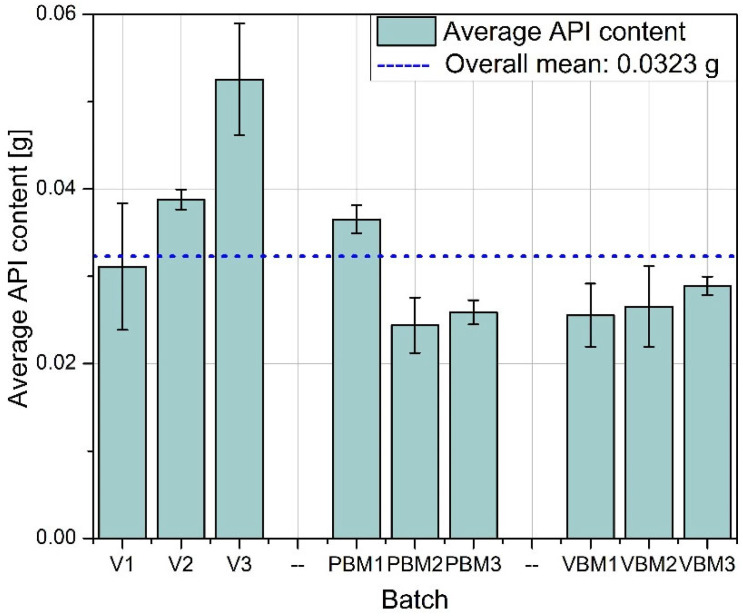
Average API content.

**Figure 15:**
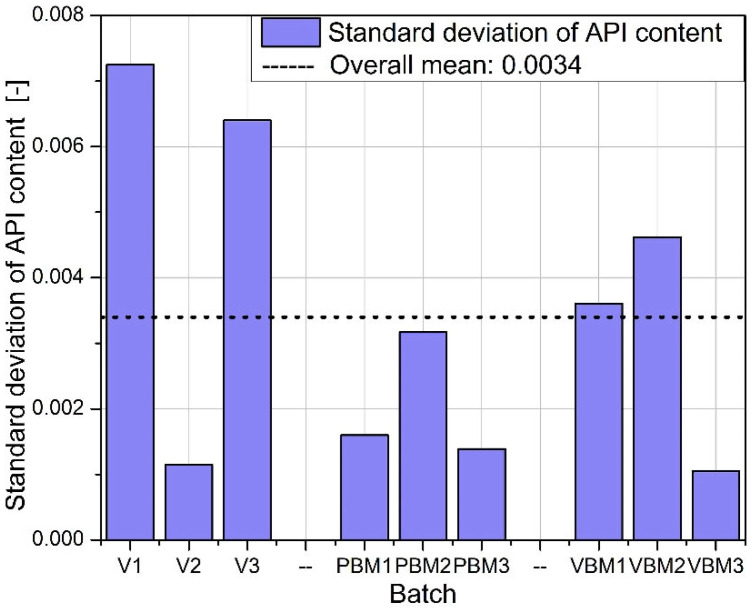
Standard deviation of API content.

Quantitative determination of the API in tablet samples, conducted via HPLC, revealed distinct differences in content uniformity across the tested mixing technologies (Figure 13, Figure 14 and Figure 15). The results underscore the influence of the blending method on the homogeneity of API distribution in final dosage forms.

Among all groups, tablets produced using the vibratory ball mill (VBM1–VBM3) demonstrated the lowest variability in API content. Individual measurements across these batches were closely clustered, indicating consistent drug distribution within the powder blend and uniformity during the compression process. This outcome aligns with previous findings on improved flow and packing behavior for VBM samples and suggests effective homogenization under vibratory mixing conditions. In contrast, tablets from the V-type mixer (V1–V3) showed the highest variability in API content. The spread of measured concentrations was notably wider compared to other groups, with differences evident both between and within batches. This inconsistency likely results from insufficient blending intensity in the V-type system, particularly in formulations containing poorly flowing or cohesive components. The findings correspond with earlier observations of high compressibility indices and limited powder mobility in these blends. The planetary ball mill samples (PBM1–PBM3) displayed intermediate performance. While some improvement in homogeneity was observed relative to the V-type batches, variability in API content remained pronounced, especially in PBM2 and PBM3. These results may reflect a balance between enhanced mixing efficiency and the onset of segregation effects induced by excessive mechanical energy. Overall, the API content data confirm that the mixing technique plays a critical role in determining dosage uniformity. Vibratory milling proved most effective in achieving homogeneous blends, while low-energy V-type mixing was associated with inadequate API distribution. These findings reinforce the need to match blending intensity to formulation characteristics and to control process parameters closely when using high- or low-shear mixing methods.

### 2.5. Statistical Evaluation

An analysis of variance (ANOVA) was conducted to assess the significance of differences among the three mixing methods with respect to the physicochemical parameters of the tablets, including weight, thickness, hardness, friability, and API content. Separate analyses were performed for each mixing method: the V-type blender, the planetary ball mill, and the vibratory ball mill.

The following statistical parameters were used in the analysis: DF (degrees of freedom), sum of squares, mean square, F value, prob > F, and R-square. DF indicates the number of independent values that can vary in the analysis. The sum of squares measures the total variability in the data attributable to different sources. The mean square is calculated as the sum of squares divided by its corresponding degrees of freedom and represents the average variation. The F value is the ratio of the variance explained by the model to the variance within groups, serving as a test statistic to determine significance. Prob > F denotes the probability that the observed F value could occur by chance, indicating statistical significance when below a chosen threshold (e.g., 0.05). R-square reflects the proportion of total variability in the response variable explained by the model, serving as a measure of goodness of fit.

For the V-type blender, statistically significant differences between groups were observed for tablet weight (F = 28.19; prob > F = 3.08 × 10^−9^), thickness (F = 124.67; prob > F = 0), friability (F = 21.13; prob > F = 0.0004), and API content (F = 35.18; prob > F = 1.98 × 10^−7^). Differences in tablet hardness were not statistically significant (F = 3.07; prob > F = 0.066).

In the case of the planetary ball mill, the mixing method had a significant effect on tablet thickness (F = 12.81; prob > F = 2.54 × 10^−5^), hardness (F = 25.11; prob > F = 1.65 × 10^−6^), friability (F = 2882.83; prob > F = 2.33 × 10^−13^), and API content (F = 88.15; prob > F = 8.76 × 10^−12^). No significant effect of this method was found on tablet weight (F = 0.55; prob > F = 0.581).

For the vibratory ball mill, significant differences between groups were observed for all analyzed parameters except for API content. Significant differences were found for weight (F = 225.03; prob > F = 0.0001), thickness (F = 185.95; prob > F = 0.0001), hardness (F = 8.20; prob > F = 0.00165), and friability (F = 6.34; prob > F = 0.0191), whereas differences in API content were not statistically significant (F = 2.26; prob > F = 0.126).

These results confirm that the choice of mixing method has a significant impact on tablet quality, particularly regarding thickness, friability, and API content. Detailed statistical values are presented in Table 4.

Tukey’s test was performed, taking into account both *p*-values and confidence intervals (Table 5). MeanDiff refers to the average difference between groups, and *p*-value indicates whether this difference is statistically significant. Alpha is the significance level used to assess the *p*-value. Sig shows whether the result is significant (1) or not (0). LCL (Lower Confidence Limit) and UCL (Upper Confidence Limit) define the range in which the true mean difference is expected to lie with confidence. The results confirm the statistical significance of the observed differences for most parameters, depending on the mixing method applied. The analysis demonstrates that the type of mixing affects tablet quality. For the V-type blender, significant differences were observed in weight, thickness, friability, and API content, whereas hardness did not show significant variation. In the planetary ball mill, thickness, hardness, friability, and API content were significantly affected, while weight remained unchanged. In the vibratory ball mill, significant differences were noted in weight, thickness, and hardness; friability varied only to a limited extent, and API content did not exhibit significant differences.

## 3. Discussion

The findings of this study clearly demonstrate that the choice of mixing technology has a profound influence on the critical quality attributes (CQAs) of directly compressed naproxen sodium tablets. Specifically, differences were observed in powder blend behavior, tablet mass and thickness uniformity, mechanical strength, and API content homogeneity. These effects stem not only from the energy and motion imparted during mixing but also from how well each technique accommodates the inherent flow and packing limitations of the component materials.

### 3.1. Influence of Mixing Method on Powder Blend Properties

Powder blends prepared with the V-type mixer showed the poorest flow and compressibility profiles, as evidenced by their high compressibility indices (>43%), elevated Hausner ratios (~1.78), and angles of repose exceeding 41°. These results are consistent with the well-established limitations of low-shear tumbling mixers when handling cohesive materials such as sodium naproxen or calcium carbonate [13]. Nevertheless, despite the suboptimal blend characteristics, tablets produced from V-type mixtures exhibited the most consistent performance in terms of mass and thickness, confirming previous observations that low-shear blending, when paired with compatible excipients, can ensure acceptable performance in direct compression formulations [8]. Planetary and vibratory ball mills significantly improved compressibility indices (as low as 33.7% in VBM3) and reduced Hausner ratios to ~1.51, attributed to increased particle–particle interactions due to size reduction. These findings support literature showing that mechanical energy from high-shear mixers can densify blends and improve apparent flow [9]. However, this improvement was often offset by increased blend instability, as indicated by higher angles of difference (up to 25.6° in VBM3), suggesting the onset of segregation or uncontrolled particle agglomeration.

### 3.2. Impact on Tablet Uniformity and Mechanical Properties

V-type mixing yielded tablets with low variability in mass and thickness, which is essential for dosage accuracy and regulatory compliance. These results are supported by previous studies, such as Marianni et al. (2021), who demonstrated that tumbling mixers—despite limited energy input—can produce homogenous blends when component compatibility is high and mixing time is sufficient [8]. In contrast, tablets produced from high-energy blends, particularly PBM3 and VBM2, exhibited greater variability in both weight and thickness. In contrast, tablets produced from high-energy blends, particularly PBM3 and VBM2, showed greater variability in both weight and thickness. This inconsistency requires careful control of mechanical energy during mixing to prevent mixing, particle abrasion or local over-processing in high-dose formulations. Interestingly, the highest hardness values were obtained from VBM batches, confirming that high-energy mixing enhances compactability. However, this was not always accompanied by improved content uniformity, indicating that improved mechanical properties do not guarantee adequate API distribution.

### 3.3. API Content Uniformity and Analytical Performance

The greatest differences between mixing methods were observed in the homogeneity of API content. VBM samples consistently showed the lowest RSD values (<1.5%), while V-type batches sometimes exceeded 3% variability, and excessive energy input in PBM2 and PBM3 led to increased heterogeneity, likely due to particle migration or electrostatic effects. Detection by bypass HPLC proved both rapid and accurate, mirroring the success of other simplified approaches: Şener et al. achieved an RSD of ≈2.2% for naproxen using FIA-UV [15], Stefano et al. demonstrated high-throughput simultaneous detection of naproxen and paracetamol using flow-injection amperometry [18], and Puttanapitak et al. fully resolved naproxen on a 10 × 4 mm C18 column in less than 5 min [19]. Taken together, these observations underscore that precise control of mixing parameters—and the use of streamlined but robust analytical tools, from bypass HPLC to chemometric signal separation to stability testing and thermal analysis—is essential for achieving high API homogeneity and effective quality control of pharmaceutical products.

### 3.4. Mechanistic Analysis

The differences observed between the mixing technologies can be explained by the underlying physical mechanisms governing each process, particularly particle–particle interactions and the balance between mechanical forces and powder properties.

The V-type tumble mixer operates through low-shear, gravity-driven tumbling, allowing particles to mix primarily by diffusion and gentle collision. This gentle process avoids significant particle breakage, electrostatic charging, and localized heating. Despite blends showing relatively poor flowability due to cohesive components like sodium naproxen and calcium carbonate, the V-mixer still yielded tablets with stable mass and thickness. This suggests that in systems with compatible particle sizes and densities, even low-shear mixing can achieve acceptable homogeneity for direct compression, as the absence of strong mechanical forces helps avoid segregation caused by fines or density differences.

In contrast, high-energy methods such as planetary ball milling (PBM) and vibratory ball milling (VBM) impart substantial mechanical energy through impact, friction, and shear. These forces promote particle deagglomeration and size reduction, which can be beneficial for improving blend homogeneity, particularly for cohesive APIs. This mechanistic behavior explains why vibratory ball milling produced the highest tablet hardness and the most consistent API content distribution in our study. The frequent collisions and oscillatory motion enhance the dispersion of API particles across the excipient matrix, leading to more uniform drug distribution even if the bulk blend retains poor macroscopic flow properties.

However, excessive energy input can have adverse consequences. In PBM3 and VBM2, higher variability in tablet mass, thickness, and API content was observed. Mechanistically, aggressive milling leads to the generation of fine particles, increasing cohesive forces and promoting electrostatic charging. Electrostatic effects cause particles to adhere to equipment surfaces or each other, leading to localized zones of varying composition. Local overprocessing may result in heterogeneous energy transfer within the powder bed, creating bimodal particle size distributions where fine particles coexist with larger fragments. This can promote segregation during transport, die filling, or compaction. Additionally, high surface area from fine particles increases moisture uptake, forming liquid bridges between particles and further promoting agglomeration or blend instability.

Although sodium naproxen is chemically stable under moderate stress, high mechanical energy could potentially induce changes in crystalline structure, polymorphism, or create amorphous regions in more sensitive APIs, altering solubility, flow, or compressibility.

A critical factor influencing blend behavior in our formulation is the high proportion of calcium carbonate (68%). This excipient exhibits poor flowability, high density, and small particle size, which can dominate the overall bulk properties of the blend. Its presence increases the risk of segregation due to percolation effects, where heavier particles migrate downward during vibration or handling, leading to inconsistent die filling and tablet weight variability. High calcium carbonate content may also mask the flow-improving effects of excipients like PVP or cellulose. Therefore, adjusting the proportion of calcium carbonate or using flow aids may be necessary to enhance blend uniformity in formulations with similar composition.

Taken together, these observations underline that achieving uniform blends in direct compression depends on matching the mechanical energy input with the specific physicochemical properties of both the API and excipients. While high-energy processes can be advantageous for improving dispersion in cohesive powders, they also pose risks of segregation, electrostatic charging, and possible degradation, especially in formulations dominated by poorly flowing materials like calcium carbonate.

### 3.5. Practical Implications

From a formulation development perspective, this study confirms that low-energy mixers like the V-type provide more predictable physical performance, especially when excipient compatibility is high. Conversely, while vibratory and planetary mills offer improved particle dispersion and flow, their application should be carefully tuned to avoid overprocessing or blend instability. These findings underscore the importance of a case-specific approach to mixer selection, particularly in formulations involving challenging APIs such as sodium naproxen. Furthermore, the implementation of rapid analytical techniques—such as bypass HPLC—may reduce analytical burden in early-stage development or in resource-limited environments, provided that matrix simplicity and signal clarity are assured.

### 3.6. Scale-Up Considerations

The results of this study have relevance for industrial production, although certain limitations exist when translating laboratory-scale findings to larger scales. The vibratory ball mill (VBM) demonstrated excellent results in terms of API content uniformity and tablet hardness in small batches; however, scaling up VBM is not straightforward. Industrial-scale vibratory mills are less common, and achieving uniform energy distribution in larger powder volumes may pose challenges, potentially leading to uneven mixing. In practice, manufacturers might consider high-shear mixers or continuous blending systems to replicate similar deagglomeration and dispersion effects at larger scales, but such alternatives would require careful optimization to avoid excessive mechanical stress or heat generation.

Regarding analytical methods, the bypass HPLC approach offers significant speed and simplicity for content uniformity testing, especially in simple formulations like the one studied here. However, in GMP environments, full chromatographic separation is often required to detect impurities and degradation products. While bypass HPLC may not fully replace conventional methods for impurity profiling, it could be valuable as a rapid in-process control (IPC) tool to monitor blend uniformity or tablet content during manufacturing, helping reduce analysis times and support real-time release strategies where appropriate.

## 4. Materials and Methods

### 4.1. Materials

The formulation consisted of sodium naproxen as the active pharmaceutical ingredient and four commonly used excipients, all obtained from recognized suppliers. Specifically, we sourced sodium naproxen from Divi’s Laboratories (Hyderabad, India); calcium carbonate from Chmpur (Piekary Śląskie, Poland); microcrystalline cellulose from FMC BioPolymer (Philadelphia, PA, USA); and both polyvinylpyrrolidone and magnesium stearate from Sigma-Aldrich (St. Louis, MO, USA). All components were of analytical-grade purity. The composition of the blend, expressed as mass percentages, included 20% sodium naproxen, 68% calcium carbonate serving as the primary filler, 7% microcrystalline cellulose functioning as both a filler and disintegrant, 3% polyvinylpyrrolidone acting as a binder, and 2% magnesium stearate employed as a lubricant.

### 4.2. Methods for Determining the Properties of Raw Materials and Tablet Blends

The properties of both raw powders and formulation blends were assessed using a powder testing instrument compliant with current pharmacopoeial guidelines. All analyses were carried out using the PT-S Powder Tester manufactured by Hosokawa Micron B.V. (Doetinchem, The Netherlands). The scope of the assessment included measurement of the angle of repose, angle of fall, angle of spatula, angle of difference, as well as aerated and tapped bulk densities, Hausner ratio, and compressibility index.

Particle size distribution of the raw materials was analyzed using laser diffraction with a wet dispersion setup. The measurements were performed using a Mastersizer 2000 Hydro MU system from Malvern Instruments (Malvern, Worcestershire, UK), in accordance with the ISO 13320-1:1999 standard [27].

### 4.3. Methods of Preparing Tablet Blends and Tablets

Powder blends in the first experimental group were prepared using a V-type tumble mixer with a total chamber volume of 750 cm^3^, produced by CDK (Gliwice, Poland). Each batch was blended for 20 min at rotational speeds of 10, 20, or 30 rpm. The working chamber was loaded to 40% of its maximum capacity to ensure optimal tumbling behavior. The second group of blends was processed using a Mono Pulverisette 6 planetary ball mill (Fritsch, Idar-Oberstein, Germany). The milling system featured a 500 cm^3^ zirconium oxide chamber and was loaded with zirconium oxide balls of 5 mm diameter. The weight ratio of grinding media to powder was maintained at 8:1. Each sample was milled for 5 min at rotational speeds of 200, 300, or 400 rpm. For the third group, powder mixtures were processed using a SPEX 8000M high-energy vibratory ball mill (SPEX SamplePrep, LLC, Metuchen, NJ, USA). The apparatus was fitted with a stainless steel chamber (volume: 50 cm^3^) and operated with two stainless steel balls of 10 mm diameter. Mixing durations were set to 2.5, 5, and 10 min, with the chamber filled to 30% of its total volume.

Tablet compression was carried out on a TDP 0 manual single-punch press (LFA Machines Oxford LTD, Oxfordshire, UK). A 6 mm concave punch set was used for the process, featuring beveled edges and an upper punch equipped with a score line to facilitate tablet splitting. Each unit was formed under a compression force of 3.5 kN.

### 4.4. Methods for Determining the Properties of Tablets

Tablet weight was determined using an Ohaus Pioneer PX224 analytical balance (Greifensee, Switzerland), ensuring high measurement accuracy. Tablet thickness was assessed using a digital caliper with a resolution of 0.01 mm, with measurements taken from 20 individual tablets per batch. All determinations were performed in quadruplicate to confirm measurement repeatability. Friability was evaluated using a GUNT CE 245 drum mixer (Barsbüttel, Germany). Approximately 50 g of tablets from each formulation was placed into a 1.15 dm^3^ rotating drum, which was mounted on shafts rotating at 200 rpm for a 5 min cycle. After the test, the tablet fragments were passed through a 500 μm mesh sieve. The mass of material retained on the sieve was recorded, and friability was expressed as a percentage of the original tablet mass. Each friability test was conducted in four replicates to ensure statistical robustness. Tablet hardness was measured using a static force method on 20 tablets per batch. The measurements were performed with a hardness tester produced by CDK (Gliwice, Poland).

### 4.5. Quantitative Determination of the API in Tablets by HPLC

The quantitative assessment of the API was conducted on nine randomly selected tablets from each investigated batch. The analytical procedure employed HPLC, utilizing a pre-established calibration curve to quantify the API content. The HPLC system comprised a mobile phase reservoir, a model 1110 pump, an injection valve enabling sample introduction via a syringe into a 20 µL loop, a model 1310 column oven, a bypass-type connector, and a UV detector (model 1410) responsible for capturing the signal corresponding to the presence of the analyte. System operation and data acquisition were managed via D-7000 HSM software version 3.0 (Merck, Darmstadt, Germany), facilitating control over chromatographic performance and analytical readout.

The decision to employ a bypass connector in place of a chromatographic column stemmed from the unambiguous presence of the target compound and the lack of necessity for component separation, due to the matrix’s simplicity and absence of interfering signals. The analysis was strictly quantitative and based solely on UV detector response. The resulting chromatograms exhibited well-defined single peaks, with a total analysis time of under two minutes per sample.

Initially, standard solutions of sodium naproxen were prepared. Accurately weighed quantities of tablet-forming excipients, each containing incrementally increasing amounts of sodium naproxen, were transferred into 50 mL volumetric flasks, followed by the addition of distilled water to volume. These mixtures were agitated in a shaker for one hour to ensure complete dissolution of sodium naproxen and PVP. Subsequently, the resulting suspensions were filtered through 0.2 µm syringe filters. Analytical samples were then prepared by mixing 0.5 mL of the filtrate with 4.5 mL of methanol, yielding a total volume of 5 mL. These solutions were introduced into the HPLC system via syringe injection. Measurements were carried out at 20 °C. The mobile phase consisted of a methanol–water mixture in a 90:10 *v*/*v* ratio, with a flow rate of 1 mL/min. Each determination required approximately 2 min.

For each standard, three replicate injections were performed, and peak areas were recorded from the resulting chromatograms. The averaged peak areas were plotted against the known mass of API to construct the calibration curve.

For tablet analysis, nine tablets were randomly selected from each batch and individually weighed. Each set was transferred into 100 mL volumetric flasks, to which 50 mL of distilled water was added. These were subjected to shaking for 8 h at 120 rpm. The resulting suspensions were filtered through 0.2 µm syringe filters, and 0.5 mL of the filtrate was combined with 4.5 mL of methanol, producing 5 mL analytical samples. These were analyzed under the same conditions as the calibration standards. Based on peak areas and the calibration curve equation, the API content in each tablet was calculated.

## 5. Conclusions

This study provides a comprehensive evaluation of how different powder blending technologies—V-type mixer, planetary ball mill, and vibratory ball mill—affect the uniformity, mechanical performance, and analytical quality of naproxen sodium tablets prepared by direct compression. Despite exhibiting the least favorable flow and compressibility profiles, powder blends produced using the V-type mixer yielded tablets with the most consistent physical dimensions and acceptable API content uniformity, emphasizing the reliability of low-energy tumbling mechanisms for formulations with well-balanced excipient compositions. In contrast, high-energy mixing using planetary and vibratory mills enhanced blend densification and improved flow characteristics, yet introduced variability in tablet mass and API content, particularly at higher mixing intensities. This underlines the importance of optimizing mixing parameters to prevent segregation or overprocessing in energy-intensive systems.

Importantly, the simplified analytical method employed in this study—UV detection with bypass HPLC—proved to be a fast, accurate, and resource-efficient alternative to conventional chromatographic separation. Its successful application to naproxen sodium highlights its potential for broader use in pharmaceutical quality control, especially in settings with limited analytical infrastructure.

Taken together, the results demonstrate that both technological and analytical choices play a critical role in ensuring the quality and reproducibility of solid dosage forms. The findings contribute valuable insights into formulation development strategies and support the integration of streamlined analytical approaches aligned with current trends in pharmaceutical analysis.

In future pharmaceutical applications, the technological and analytical approaches presented in this study show considerable promise, particularly for APIs exhibiting good UV absorbance, moderate to high solubility, and low ionization variability, which favor simplified analytical techniques such as the bypass HPLC method. However, while the bypass HPLC approach was successfully validated for a relatively simple formulation, its broader applicability to more complex products requires further investigation and validation in GMP environments. From a technological perspective, low-shear mixing methods like V-type blending remain suitable for APIs and excipients with favorable flow properties and comparable bulk densities, whereas high-energy mixing techniques such as planetary or vibratory ball milling may be necessary for cohesive or poorly soluble APIs to achieve particle size reduction and improved dispersion. Nevertheless, the mechanical stress associated with high-energy processes must be carefully managed to prevent degradation, polymorphic transitions, or alterations in the physicochemical characteristics of sensitive APIs. Although this study offers valuable insights into the influence of mixing technologies on blend uniformity and tablet quality, it is important to acknowledge limitations related to the laboratory scale of the experiments and the need for further studies to confirm scalability and industrial feasibility. Future research should therefore focus on adapting both mixing intensities and analytical strategies to the diverse physicochemical profiles of different APIs, explore larger-scale processes, and expand the validation of simplified analytical methods, ultimately aiming to optimize solid dosage form production for both quality and efficiency.

## Figures and Tables

**Table 1:** Experimental plan.

Batch	Symbol
V-type mixer, 10 rpm, mixing time 20 min	V1
V-type mixer, 20 rpm, mixing time 20 min	V2
V-type mixer, 30 rpm, mixing time 20 min	V3
Planetary ball mill, 200 rpm, mixing time 5 min	PBM1
Planetary ball mill, 300 rpm, mixing time 5 min	PBM2
Planetary ball mill, 400 rpm, mixing time 5 min	PBM3
Vibrating ball mill (mixing time 2 min)	VBM1
Vibrating ball mill (mixing time 5 min)	VBM2
Vibrating ball mill (mixing time 10 min)	VBM3

**Table 2:** Raw material characteristics.

Sample	Angle of Repose	Angle of Fall	Angle of Difference	Angle of Spatula	Aerated Bulk Density	Packed Bulk Density	Compressibility	Hausner Ratio
	[deg]	[deg]	[deg]	[deg]	[g/cm^3^]	[g/cm^3^]	[%]	[-]
Sodium naproxen	46.1	23.2	22.9	67.2	0.380	0.692	45.1	1.82
Polyvinylpyrrolidone (PVP)	39.9	16.7	23.2	48.3	0.425	0.509	16.5	1.20
Magnesium stearate	39.7	19.4	20.5	63.2	0.379	0.581	34.8	1.53
Cellulose	44.2	23.7	20.5	46.1	0.398	0.488	18.4	1.23
Calcium carbonate	43.1	29.7	13.4	69.4	0.449	0.864	48.0	1.92

**Table 3:** Mixture characteristics.

Sample	Angle of Repose	Angle of Fall	Angle of Difference	Angle of Spatula	Aerated Bulk Density	Packed Bulk Density	Compressibility	Hausner Ratio
	[deg]	[deg]	[deg]	[deg]	[g/cm^3^]	[g/cm^3^]	[%]	[-]
V1	39.6	19.2	20.4	55.4	0.418	0.749	44.2	1.79
V2	41.4	20.1	21.3	55.2	0.412	0.735	43.9	1.78
V3	42.7	19.8	22.9	55.6	0.408	0.724	43.6	1.77
PBM1	40.3	21.2	19.1	54.1	0.421	0.722	41.7	1.71
PBM2	39.4	18.7	20.7	53.8	0.428	0.724	40.9	1.69
PBM3	39.1	17.6	21.5	53.1	0.437	0.713	38.7	1.63
VBM1	39.4	18.4	21	54.3	0.4752	0.7164	33.7	1.51
VBM2	41	17.2	23.8	54.8	0.4818	0.7356	34.5	1.53
VBM3	40.2	14.6	25.6	53.2	0.4884	0.7386	33.9	1.51

**Table 4:** Results of the ANOVA statistical analysis.

V-Type Mixer						
	DF	Sum of Squares	Mean Square	F Value	Prob > F	R-Square
Table mass						
Model	2	0.00275	0.00137	28.19071	3.08 × 10^−9^	0.49727
Error	57	0.00278	4.87 × 10^−5^			
Total	59	0.00552				
Tablet thickness						
Model	2	0.66449	0.33225	124.67452	0	0.81394
Error	57	0.1519	0.00266			
Total	59	0.81639				
Tablet hardness						
Model	2	1.98 × 10^−4^	9.88 × 10^−5^	3.06757	0.06593	0.21058
Error	23	7.41 × 10^−4^	3.22 × 10^−5^			
Total	25	9.38 × 10^−4^				
Tablet friability						
Model	2	2.41 × 10^−4^	1.21 × 10^−4^	21.13236	3.98 × 10^−4^	0.82444
Error	9	5.13 × 10^−5^	5.7 × 10^−6^			
Total	11	2.92 × 10^−4^				
API content						
Model	2	0.0018	8.99 × 10^−4^	35.17789	1.98 × 10^−7^	0.77013
Error	21	5.37 × 10^−4^	2.56 × 10^−5^			
Total	23	0.00233				
Planetary ball mill						
Tablet mass						
Model	2	8.62 × 10^−5^	4.31 × 10^−5^	0.54859	0.58078	0.01889
Error	57	0.00448	7.86 × 10^−5^			
Total	59	0.00457				
Tablet thickness						
Model	2	0.20645	0.10323	12.81286	2.54 × 10^−5^	0.31014
Error	57	0.45922	0.00806			
Total	59	0.66567				
Tablet hardness						
Model	2	0.00442	0.00221	25.10942	1.65 × 10^−6^	0.68587
Error	23	0.00202	8.8 × 10^−5^			
Total	25	0.00644				
Tablet friability						
Model	2	0.14095	0.07048	2882.8255	2.33 × 10^−13^	0.99844
Error	9	2.2 × 10^−4^	2.44 × 10^−5^			
Total	11	0.14117				
API content						
Model	2	8.16 × 10^−4^	4.08 × 10^−4^	88.14795	8.76 × 10^−12^	0.88018
Error	24	1.11 × 10^−4^	4.63 × 10^−6^			
Total	26	9.27 × 10^−4^				
Vibrating ball mill						
Tablet mass						
Model	2	0.03505	0.01752	225.02656	0	0.88759
Error	57	0.00444	7.79 × 10^−5^			
Total	59	0.03949				
Tablet thickness						
Model	2	2.83496	1.41748	185.95486	0	0.8671
Error	57	0.43449	0.00762			
Total	59	3.26946				
Tablet hardness						
Model	2	3.67 × 10^−4^	1.83 × 10^−4^	8.20131	0.00165	0.37792
Error	27	6.04 × 10^−4^	2.24 × 10^−5^			
Total	29	9.71 × 10^−4^				
Tablet friability						
Model	2	2.68 × 10^−4^	1.34 × 10^−4^	6.34229	0.01912	0.58496
Error	9	1.90 × 10^−4^	2.11 × 10^−5^			
Total	11	4.59 × 10^−4^				
API content						
Model	2	3.83 × 10^−5^	1.92 × 10^−5^	2.26401	0.12568	0.15872
Error	24	2.03 × 10^−4^	8.47 × 10^−6^			
Total	26	2.42 × 10^−4^				

**Table 5:** Tukey’s post hoc test results.

V-Type Mixer						
	MeanDiff	*p*-Value	Alpha	Sig	LCL	UCL
Tablet mass						
V2 V1	0.00695	0.00723	0.05	1	0.00164	0.01226
V3 V1	0.0165	0	0.05	1	0.01119	0.02181
V3 V2	0.00955	1.79 × 10^−4^	0.05	1	0.00424	0.01486
Tablet thickness						
V2 V1	0.021	0.4086	0.05	0	−0.01828	0.06028
V3 V1	0.233	0	0.05	1	0.19372	0.27228
V3 V2	0.212	0	0.05	1	0.17272	0.25128
Tablet hardness						
V2 V1	0.00255	0.66399	0.05	0	−0.00479	0.00989
V3 V1	−0.00371	0.42782	0.05	0	−0.01105	0.00363
V3 V2	−0.00626	0.05402	0.05	0	−0.01262	9.44 × 10^−5^
Tablet friability						
V2 V1	0.00912	0.00112	0.05	1	0.00441	0.01384
V3 V1	−0.000725	0.90445	0.05	0	−0.00544	0.00399
V3 V2	−0.00985	6.52 × 10^−4^	0.05	1	−0.01457	−0.00513
API content						
V2 V1	0.00634	0.06661	0.05	0	−3.78 × 10^−4^	0.01305
V3 V1	0.02095	2.87 × 10^−7^	0.05	1	0.01423	0.02766
V3 V2	0.01461	1.27 × 10^−5^	0.05	1	0.0086	0.02062
Planetary ball mill						
Tablet mass						
PBM2 PBM1	−3.5 × 10^−4^	0.99145	0.05	0	−0.0071	0.0064
PBM3 PBM1	0.00235	0.68099	0.05	0	−0.0044	0.0091
PBM3 PBM2	0.0027	0.60299	0.05	0	−0.00405	0.00945
Tablet thickness						
PBM2 PBM1	−0.142	1.69 × 10^−5^	0.05	1	−0.2103	−0.0737
PBM3 PBM1	−0.09	0.00681	0.05	1	−0.1583	−0.0217
PBM3 PBM2	0.052	0.16841	0.05	0	−0.1583	0.1203
Tablet hardness						
PBM2 PBM1	0.03427	9.12 × 10^−7^	0.05	1	0.02214	0.0464
PBM3 PBM1	0.023	2.49 × 10^−4^	0.05	1	0.01087	0.03513
PBM3 PBM2	−0.01127	0.03392	0.05	1	−0.02178	−7.64 × 10^−4^
Tablet friability						
PBM2 PBM1	0.22877	0	0.05	1	0.21901	0.23854
PBM3 PBM1	0.23102	0	0.05	1	0.22126	0.24079
PBM3 PBM2	0.00225	0.8005	0.05	0	−0.00751	0.01201
API content						
PBM2 PBM1	−0.01231	0	0.05	1	−0.01484	−0.00978
PBM3 PBM1	−0.01231	0	0.05	1	−0.01342	−0.00836
PBM3 PBM2	0.00142	0.35704	0.05	0	−0.00111	0.00395
Vibrating ball mill						
Tablet mass						
VBM2 VBM1	0.01635	7.03 × 10^−7^	0.05	1	0.00963	0.02307
VBM3 VBM1	0.05745	0	0.05	1	0.05073	0.06417
VBM3 VBM2	0.0411	0	0.05	1	0.03438	0.04782
Tablet thickness						
VBM2 VBM1	0.0325	0.47155	0.05	0	−0.03394	0.09894
VBM3 VBM1	0.4765	0	0.05	1	0.41006	0.54294
VBM3 VBM2	0.444	0	0.05	1	0.37756	0.51044
Tablet hardness						
VBM2 VBM1	0.0064	0.0144	0.05	1	0.00116	0.01165
VBM3 VBM1	0.00813	0.00187	0.05	1	0.00289	0.01337
VBM3 VBM2	0.00173	0.69588	0.05	0	−0.00352	0.00697
Tablet friability						
VBM2 VBM1	0.00252	0.72591	0.05	0	−0.00655	0.0116
VBM3 VBM1	0.01105	0.01949	0.05	1	0.00197	0.02013
VBM3 VBM2	0.00853	0.06517	0.05	0	−5.53 × 10^−4^	0.0176
API content						
VBM2 VBM1	0.00104	0.73	0.05	0	−0.00238	0.00447
VBM3 VBM1	0.00288	0.11073	0.05	0	−5.43 × 10^−4^	0.00631
VBM3 VBM2	0.00184	0.38733	0.05	0	−0.00159	0.00526

## Data Availability

The data that support the findings of this study are available from the corresponding author upon reasonable request.

## References

[1] Kudo Y., Yasuda M., Matsusaka S. (2020). Effect of particle size distribution on flowability of granulated lactose. Adv. Powder Technol..

[2] Przywara M., Leszczak P. (2025). Quality by design strategies in fluidized bed granulation: A focus on granules for tablet manufacturing. Adv. Sci. Technol. Res. J..

[3] Nurjanah A., Nurlelah N. (2023). Formulation and Physical Evaluation of CTM Tablets by Direct Compression Method: A Systematic Literature Review. Eureka Herba Indones..

[4] Sulaiman T.N.S., Sulaiman S. (2020). Review: Excipients for Tablet Manufacturing With Direct Compression Method. J. Pharm. Sci..

[5] Iqubal M.K., Singh P.K., Shuaib M., Iqubal A., Singh M. (2014). Recent Advances in Direct Compression Technique for Pharmaceutical Tablet Formulation. Int. J. Pharm. Res. Dev..

[6] Jivraj M., Martini L.G., Thomson C.M. (2000). An overview of the different excipients useful for the direct compression of tablets. Pharm. Sci. Technol. Today.

[7] Gohel M.C., Jogani P.D. (2005). A review of co-processed directly compressible excipients. J. Pharm. Pharm. Sci..

[8] Marianni B., Polonini H., Oliveira M.A.L. (2021). Ensuring homogeneity in powder mixtures for pharmaceuticals and dietary supplements: Evaluation of a 3-axis mixing equipment. Pharmaceutics.

[9] Asachi M., Nourafkan E., Hassanpour A. (2018). A review of current techniques for the evaluation of powder mixing. Adv. Powder Technol..

[10] Przywara M., Jękot K., Jednacz W. (2025). Evaluation of Vibratory Ball Mill Mixing as an Alternative to Wet Granulation in the Manufacturing of Sodium Naproxen Tablets with Dolomite-Based Formulations. Appl. Sci..

[11] Leś K., Opaliński I. (2021). Prospective Application of Response Surface Methodology for Predicting High-Energy Mixing Process Conditions towards Fine Powders Flow Improvement. Adv. Sci. Technol. Res. J..

[12] Villiers M.M., Tiedt L.R. (1996). An analysis of fine grinding and aggregation of poorly soluble drug powders in a vibrating ball mill. Pharmazie.

[13] Jakubowska E., Ciepluch N. (2021). Blend segregation in tablets manufacturing and its effect on drug content uniformity—A review. Pharmaceutics.

[14] Di Martino P., Malaj L., Censi R., Martelli S. (2008). Physico-chemical and technological properties of sodium naproxen granules prepared in a high-shear mixer-granulator. J. Pharm. Sci..

[15] Sener E., Tuncel M., Aboul-Enein H.Y. (2003). Rapid determination of naproxen sodium in pharmaceutical formulations by flow injection analysis (FIA) using UV-detection. J. Liq. Chromatogr. Relat. Technol..

[16] Maghsoodi M., Taghizadeh O., Martin G.P., Nokhodchi A. (2008). Particle design of naproxen-disintegrant agglomerates for direct compression by a crystallo-co-agglomeration technique. Int. J. Pharm..

[17] Wang L.J., Tang Y.H., Liu Y.H. (2011). Flow injection chemiluminescence determination of loxoprofen and naproxen with the acidic permanganate-sulfite system. J. Pharm. Anal..

[18] Stefano J.S., Montes R.H.O., Richter E.M., Muñoz R.A.A. (2014). Flow-injection analysis with multiple-pulse amperometry for simultaneous determination of paracetamol and naproxen using a homemade flow cell for screen-printed electrodes. J. Braz. Chem. Soc..

[19] Puttanapitak P., Chokesinlapasart S., Suntornsuk L., Phechkrajang C., Prutthiwanasan B. (2022). A simple and rapid chromatographic method for determination of naproxen and nabumetone in tablets. Sep. Sci. Plus.

[20] Dinç E., Özdemir A., Aksoy H., Üstündaǧ Ö., Baleanud D. (2006). Chemometric determination of naproxen sodium and pseudoephedrine hydrochloride in tablets by HPLC. Chem. Pharm. Bull..

[21] Pushpa Latha E., Sailaja B. (2020). Stability indicating rp-hplc method development and validation for the determination of naproxen sodium in bulk drug and tablet dosage form. Int. J. Pharm. Qual. Assur..

[22] Ramos P., Raczak B.K., Silvestri D., Wacławek S. (2023). Application of TGA/c-DTA for Distinguishing between Two Forms of Naproxen in Pharmaceutical Preparations. Pharmaceutics.

[23] Kaynak M.S., Şahin S. (2008). A new Hplc approach for determination of in-vitro solubility of naproxen sodium. Hacettepe Univ. J. Fac. Pharm..

[24] Yang Y., Xia Y., Wei F., Teng G., Yao Y. (2020). Preparation and characterization of hydrophobic stearic acid-Yb-PbO2 anode and its application on the electrochemical degradation of naproxen sodium. J. Electroanal. Chem..

[25] Tanjin S., Islam F., Sultan M.Z., Rahman A., Chowdhury S.R., Sharmin T., Rashid M.A. (2015). Development and Validation of a Simple RP-HPLC Method for Determination of Naproxen in Pharmaceutical Dosage Forms. Bangladesh Pharm. J..

[26] Vittal S.P., Rao S.V., Ramakrishna K. (2019). Stability indicating rp-hplc method for simultaneous determination of potential impurities of sumatriptan and naproxen sodium in fixed dose combination. Rasayan J. Chem..

[27] (1999). Particle Size Analysis—Laser Diffraction Methods—Part 1: General Principles.

